# Correction: Cysteine dioxygenase 1 is a metabolic liability for non-small cell lung cancer

**DOI:** 10.7554/eLife.52671

**Published:** 2019-10-18

**Authors:** Yun Pyo Kang, Laura Torrente, Aimee Falzone, Cody M Elkins, Min Liu, John M Asara, Christian C Dibble, Gina DeNicola

Yun Pyo Kang, Laura Torrente, Aimee Falzone, Cody M Elkins, Min Liu, John M Asara, Christian C Dibble, Gina M DeNicola Kang YP, Torrente L, Falzone A, Elkins CM, Liu M, Asara JM, Dibble CC, DeNicola GM. 2019. Cysteine dioxygenase 1 is a metabolic liability for non-small cell lung cancer. *eLife* **8**:e45572. doi: 10.7554/eLife.45572.

Published 20, May 2019

Figure 3, Figure 3—figure supplement 2B, Figure 5—figure supplement 3A and Figure 5—figure supplement 4C have an erroneous label that groups the PC9 cell line with KEAP1 mutant cell lines. This cell line, while previously characterized as having high NRF2 activity (DeNicola GM et al., Nat Genet. 2015 Dec;47(12):1475-81), do not have a KEAP1 mutation. This error does not affect our conclusions, but may lead to confusion in the field about the mutation status of this cell line, which we would like to avoid. Thus, this labelling error is being changed as following.

## Correction of figure labels (1-4): KEAP1^WT^→ NRF2^LOW^; KEAP1^MUT^→ NRF2^HIGH^

### 1. Figure 3D

#### After correction

**Figure 1:**
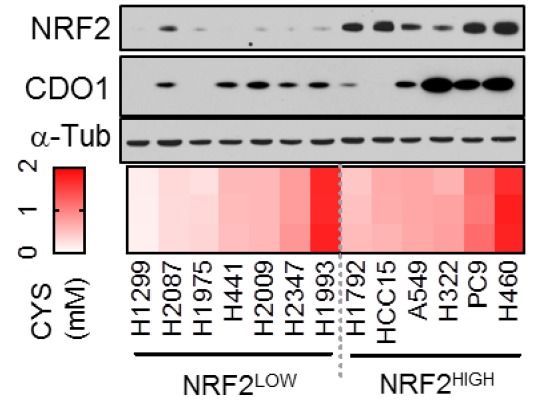


#### Before correction

**Figure 2:**
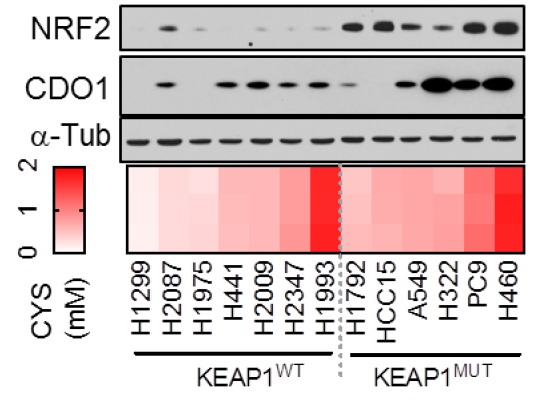


### 2. Figure 3—figure supplement 2B

#### After correction

**Figure 3:**
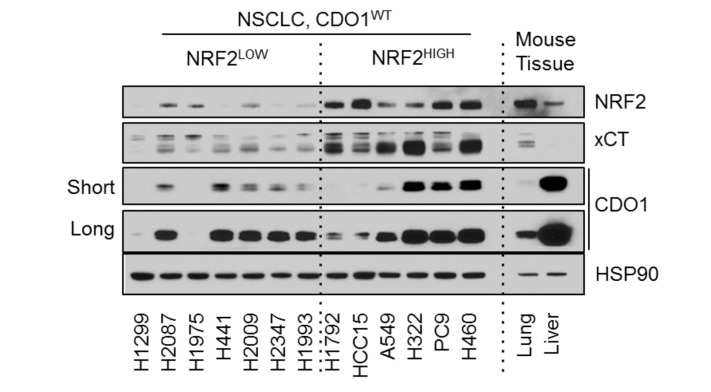


#### Before correction

**Figure 4:**
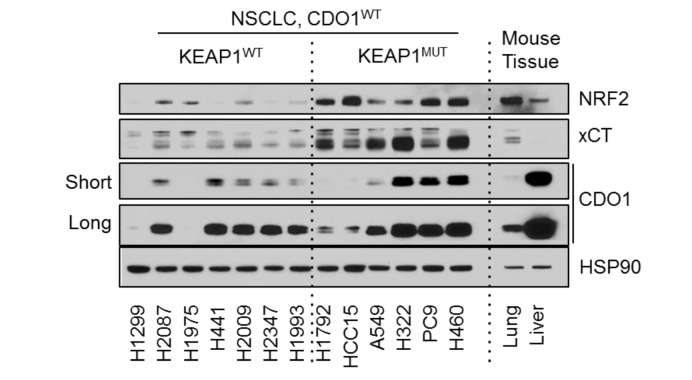


### 3. Figure 5—figure supplement 3A

#### After correction

**Figure 5:**
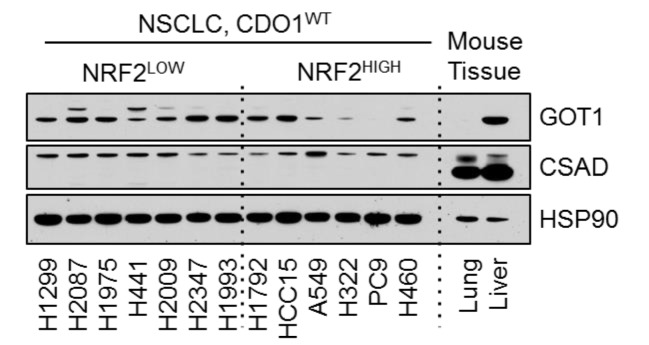


#### Before correction

**Figure 6:**
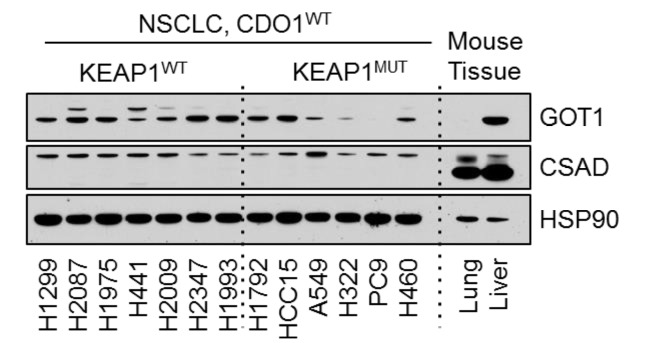


### 4. Figure 5—figure supplement 4C

#### After correction

**Figure 7:**
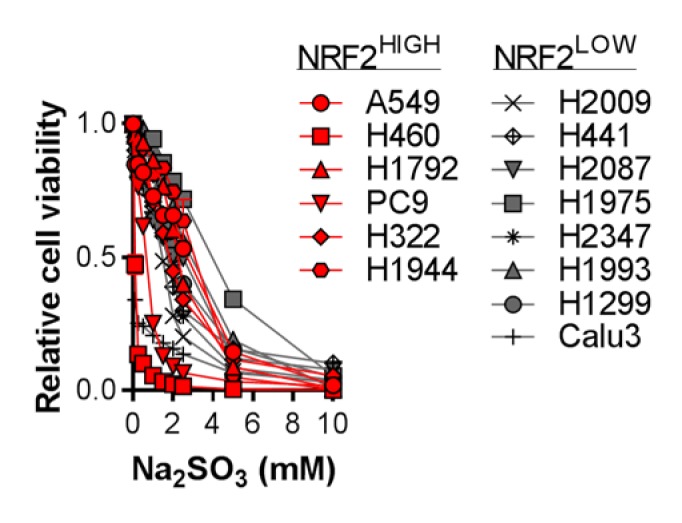


#### Before correction

**Figure 8:**
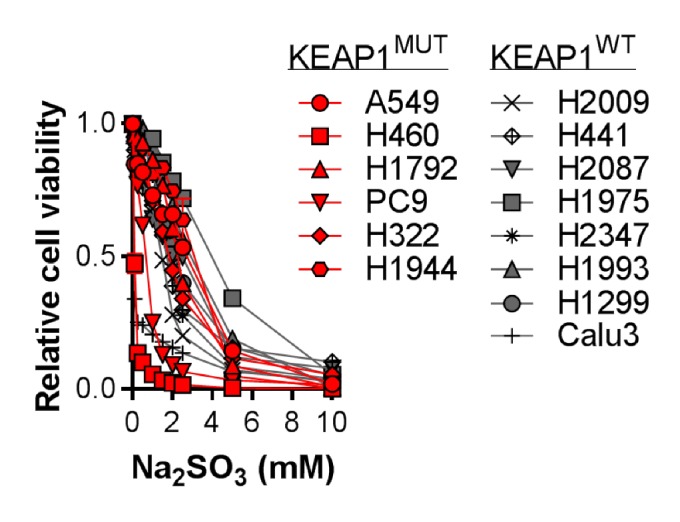


## Correction of description in figure legends (Changes are underlined):

### 1. Figure 3

#### After correction

(d) (Top) Western blot analysis of NRF2, CDO1 and α-Tubulin expression in NRF2^LOW^ and NRF2^HIGH^ (KEAP1 mutant: H1792, HCC15, A549, H322, and H460) NSCLC cell lines expressing CDO1^Y157F^. (Bottom) Intracellular cysteine concentration of the parental cell lines (minus doxycycline). (Right) Correlation between CDO1 protein levels and intracellular cysteine concentrations. CDO1 protein was normalized to α-Tubulin. N = 13.

#### Before correction

(d) (Top) Western blot analysis of NRF2, CDO1 and α-Tubulin expression in KEAP1 wild-type and KEAP1 mutant NSCLC cell lines expressing CDO1^Y157F^. (Bottom) Intracellular cysteine concentration of the parental cell lines (minus doxycycline). (Right) Correlation between CDO1 protein levels and intracellular cysteine concentrations. CDO1 protein was normalized to α-Tubulin. N = 13.

### 2. Figure 5—figure supplement 3A

#### After correction

(a) Western blot analysis of GOT1, CSAD and HSP90 levels in NRF2^LOW^ and NRF2^HIGH^ NSCLC cell lines. Mouse liver is included as a positive control for GOT1 and CSAD expression. Cells were fed with fresh RPMI +10% FBS 4 hr prior to lysate collection.

#### Before correction

(a) Western blot analysis of GOT1, CSAD and HSP90 levels in KEAP1 wild-type and KEAP1 mutant NSCLC cell lines. Mouse liver is included as a positive control for GOT1 and CSAD expression. Cells were fed with fresh RPMI +10% FBS 4 hr prior to lysate collection.

### 3. Figure 5—figure supplement 4B

#### After correction

(b) Analysis of cell viability following starvation of NRF2^LOW^ (gray) and NRF2^HIGH^ (red) NSCLC cell lines of cystine for 3 days. Cell numbers were analyzed with crystal violet and normalized to cells grown in cystine-containing media. N = 3 replicates/group.

#### Before correction

(b) Analysis of cell viability following starvation of KEAP1 wild-type (gray) and KEAP1 mutant (red) NSCLC cell lines of cystine for 3 days. Cell numbers were analyzed with crystal violet and normalized to cells grown in cystine-containing media. N = 3 replicates/group.

### 4. Figure 5—figure supplement 4C

#### After correction

(c) Analysis of cell viability following treatment of NRF2^LOW^ (gray) and NRF2^HIGH^ (red) NSCLC cell lines with 0–10 mM Na_2_SO_3_ for 3 days. Viable cells were analyzed with CellTiter-Glo and normalized to untreated cells. N = 3 replicates/group.

#### Before correction

(c) Analysis of cell viability following treatment of KEAP1 wild-type (gray) and KEAP1 mutant (red) NSCLC cell lines with 0–10 mM Na_2_SO_3_ for 3 days. Viable cells were analyzed with CellTiter-Glo and normalized to untreated cells. N = 3 replicates/group.

## Correction of description in main text (Changes are underlined):

### 1. After correction

To investigate the NRF2-dependent regulation of CDO1 protein in NSCLC, we generated a doxycycline-inducible lentiviral expression system to reintroduce GFP, CDO1^WT^ or a catalytically inactive CDO1 mutant (Y157F, Ye et al., 2007) at single copy into the panel of NRF2^LOW^ and NRF2^HIGH^ NSCLC cell lines (Figure 3D, figure 3—﻿figure supplement 2). The level of CDO1 protein expression in these cells was similar with the physiological Cdo1 levels in mouse lung and liver (Figure 3—﻿figure supplement 2B), with liver being one of the highest CDO1-expressing tissues that is responsible for supplying TAU to the body (Stipanuk et al., 2015). We find that CDO1 accumulated to higher levels in NRF2^HIGH^ cells than NRF2^LOW^, although accumulation was observed in many NRF2^LOW^ cell lines as well (Figure 3D).

### Before correction

To investigate the NRF2-dependent regulation of CDO1 protein in NSCLC, we generated a doxycycline-inducible lentiviral expression system to reintroduce GFP, CDO1^WT^ or a catalytically inactive CDO1 mutant (Y157F, Ye et al., 2007) at single copy into the panel of KEAP1^WT^ and KEAP1^MUT^ NSCLC cell lines (Figure 3D, figure 3—﻿figure supplement 2). The level of CDO1 protein expression in these cells was similar with the physiological Cdo1 levels in mouse lung and liver (Figure 3—﻿figure supplement 2B), with liver being one of the highest CDO1-expressing tissues that is responsible for supplying TAU to the body (Stipanuk et al., 2015). We find that CDO1 accumulated to higher levels in KEAP1^MUT^ cells than KEAP1^WT^, although accumulation was observed in many KEAP1^WT^ cell lines as well (Figure 3D).

### 2. After correction

Next, we examined the toxicity of CDO1 products to NSCLC cell lines. Treatment of cells with CSA and Na_2_SO_3_, but not HTAU, led to cytotoxicity (Figure 5—﻿figure supplement 4A). We found that (CYS)_2_ starvation and Na_2_SO_3_ treatment were universally toxic to NSCLC cell lines, which did not depend on NRF2 activity (Figure 5—﻿figure supplement 4B,C). In addition, CDO1, CSA and Na_2_SO_3_ sensitized A549 cells to oxidative stress (Figure 5—﻿figure supplement 4D,E), consistent with their ability to deplete (CYS)_2_. Collectively, these results demonstrate that CSA and SO_3_^2-^ are toxic to NSCLC cells regardless of NRF2 activity, suggesting that resistance to (CYS)_2_ starvation is not an inherent phenotype of NRF2^HIGH^ cells. Rather, they are sensitive to CDO1 expression due to high intracellular CYS and CDO1 stabilization.

### Before correction

Next, we examined the toxicity of CDO1 products to NSCLC cell lines. Treatment of cells with CSA and Na_2_SO_3_, but not HTAU, led to cytotoxicity (Figure 5—﻿figure supplement 4A). We found that (CYS)_2_ starvation and Na_2_SO_3_ treatment were universally toxic to NSCLC cell lines, which did not depend on KEAP1 mutation status (Figure 5—﻿figure supplement 4B,C). In addition, CDO1, CSA and Na_2_SO_3_ sensitized A549 cells to oxidative stress (Figure 5—﻿figure supplement 4D,E), consistent with their ability to deplete (CYS)_2_. Collectively, these results demonstrate that CSA and SO_3_^2-^ are toxic to NSCLC cells regardless of KEAP1 mutation status, suggesting that resistance to (CYS)_2_ starvation is not an inherent phenotype of KEAP1 mutant cells. Rather, they are sensitive to CDO1 expression due to high intracellular CYS and CDO1 stabilization.

The article has been corrected accordingly.

